# Regulation of the Functions of Natural Cytotoxicity Receptors by Interactions with Diverse Ligands and Alterations in Splice Variant Expression

**DOI:** 10.3389/fimmu.2017.00369

**Published:** 2017-03-30

**Authors:** Tatiana Pazina, Avishai Shemesh, Michael Brusilovsky, Angel Porgador, Kerry S. Campbell

**Affiliations:** ^1^Blood Cell Development and Function Program, Institute for Cancer Research, Fox Chase Cancer Center, Philadelphia, PA, USA; ^2^Federal State Budgetary Scientific Institution “Institute of Experimental Medicine”, St. Petersburg, Russia; ^3^Faculty of Health Sciences, The Shraga Segal Department of Microbiology, Immunology and Genetics, Ben-Gurion University of the Negev, Beer Sheva, Israel; ^4^Division of Allergy and Immunology, Cincinnati Children’s Hospital Medical Center, Cincinnati, OH, USA

**Keywords:** natural cytotoxicity receptors, natural killer cells, RNA splice variants, cytotoxicity, cancer immunology, virus immunity, human immunology, pregnancy

## Abstract

The natural cytotoxicity receptor (NCR) family is constituted by NKp46, NKp44, and NKp30 in humans, which are expressed mainly on natural killer (NK) cells and are encoded by the *ncr1, ncr2*, and *ncr3* genes, respectively. NCRs have classically been defined as activating receptors that trigger cytotoxicity and cytokine responses by NK cells upon engaging with ligands on tumor cells. Several new findings, however, have challenged this model and identified alternative mechanisms regulating the function of NCRs. Recent reports indicate that ligand matters, since the interaction of NKp44 with distinct ligands on target cells can either activate or inhibit NK cells. Also, the NCRs have been found to interact with distinct specificities to various heparan sulfate glycosaminoglycans, which are complex polysaccharides found in extracellular matrix or on cell surface heparan sulfate proteoglycans (HSPGs). The NCRs can engage with HSPGs in trans as a co-ligand on the target cells or in cis on the NK cell surface to regulate receptor–ligand interactions and NK cell activation. A number of splice variants of *ncr2* and *ncr3* have also been identified, and a predominant expression of certain variants results in inhibitory signaling through NKp44 and NKp30. Several recent studies have found that the selective expression of some of these inhibitory splice variants can significantly influence outcome in the contexts of cancer, infection, and pregnancy. These findings establish that NCR functions are more diverse than originally thought, and better understanding of their splice variant expression profiles and ligand interactions are needed to establish their functional regulation in the context of human health.

## Introduction

It has become increasingly clear that human natural killer (NK) cells use an array of germline-encoded cell surface receptors to spontaneously recognize and respond to the abnormal status of tumor cells, virus-infected cells, and stressed cells (1). Different NK cell surface receptors transduce either activating or inhibitory signals, directly or through associated adaptor proteins, to dynamically regulate the activation state of NK cells (2–4). Inhibitory receptors, such as killer cell Ig-like receptors (KIRs) and CD94/NKG2A, provide NK cells with a dominant, tolerizing immune checkpoint through their recognition of the MHC class I (MHC-I) molecules ubiquitously expressed on the surfaces of most normal cells. The loss of MHC-I by many transformed cells, however, overcomes this inhibitory checkpoint to favor activation. The loss of inhibitory restraint allows activating receptor signals to predominate and triggers exocytosis of perforin and granzymes to induce targeted apoptosis of the MHC-I-deficient cell and localized secretion of pro-inflammatory cytokines [especially interferon (IFN)-γ, TNF-α, and several chemokines].

The activation signals transduced in NK cells are derived from adhesion molecules (especially LFA-1), co-stimulatory receptors (such as NKG2D, DNAM-1, and SLAM family receptors), and several activating receptors physically linked to the immunoreceptor tyrosine-based activation motif (ITAM)-containing transmembrane proteins, DAP12, TCR-ζ, and/or FcεRI-γ (2–4). The key ITAM-coupled activating receptors on human NK cells, include CD16 (FcγRIIIa), an activating subfamily of KIR (KIR2DS or KIR3DS receptors), CD94/NKG2C, and the natural cytotoxicity receptors (NCRs).

The human NCRs consist of three receptors, named NKp46 (NCR1, CD335), NKp44 (NCR2, CD336), and NKp30 (NCR3, CD337). These NCR were classically defined as germline-encoded receptors that play important roles in the activation of human NK cells toward transformed target cells (5, 6). Recent work, however, has established that the NCRs can also generate inhibitory responses under certain circumstances. Here, we will review our current understanding of the expression and function of NCRs on NK cells, particularly in humans, although it is important to note that the NCRs are also expressed on other innate lymphoid cells (ILCs) and a subset of T cells, which has been previously reviewed elsewhere (7, 8).

## NCRs and Their Structures

The NCRs were initially discovered and characterized by the laboratories of Alessandro and Lorenzo Moretta in the late 1990s (9–13). They are type I transmembrane glycoproteins that were originally recognized as activating receptors and named in accordance with their molecular weight on SDS-PAGE (NKp30, NKp44, and NKp46). NKp46 is the only NCR also expressed in mice, although a receptor analogous to NKp30 has been shown to be expressed in 1 of 13 mouse strains examined (14) and in rats (15, 16). While the *ncr2* and *ncr3* genes encoding NKp44 and NKp30, respectively, are localized to human MHC class III locus on chromosome 6, the NKp46 encoding gene, *ncr1*, is found near the leukocyte regulatory complex on human chromosome 19 (10, 12, 13).

NKp46 has been shown to be a highly selective marker of all NK cells in mouse and man, although surface expression can be low on some NK cells, particularly in humans, and the receptor is also expressed on some ILCs and a small subset of T cells (9, 17, 18). Importantly, NKp46 is not expressed by CD1d-restricted invariant NKT cells in mice and humans (17). NKp46 has been shown to provide NK cells with the capacity to recognize and kill a variety of tumor target cells (19–21). NKp46 ligands have been reported to be enriched in areas of high malignant potential and high proliferation within melanoma lesions, whereas surrounding normal melanocytes were found to lack NKp46 ligands (22). Evidence in mice also suggests that NKp46 also contributes to the development of type 1 diabetes by interacting with an uncharacterized ligand on pancreatic islet beta cells (21). As a tumor immunosuppressive mechanism, the surface expression of NKp46 on NK cells can be down-modulated by exposure to l-kynurenine, which is a tryptophan catabolism product generated by the indoleamine 2,3-dioxygenase (IDO) enzyme in tumor microenvironments (23).

NKp30, similar to NKp46, is expressed on nearly all human NK cells (13). This NCR has been shown to play important roles in crosstalk between NK cells and dendritic cells (DCs) through promoting both the maturation of and the cytotoxicity of immature DC (24, 25). Surface expression levels of NKp30 and NKp46 can be upregulated by IFN-α, IL-2, and prolactin and downregulated by cortisol and methylprednisolone (26–28). In addition, both receptors are also commonly downregulated in “adaptive” or “memory-like” NK cells that are found in some cytomegalovirus-infected individuals (29, 30). TGF-β has been shown to selectively down-modulate the expression of NKp30, but not NKp46 on NK cells (31).

NKp44 is distinct among NCRs, since it is unique to humans and only expressed constitutively on some CD56^bright^ NK cells in a subset of individuals, but expression can be upregulated on essentially all NK cells after culture with IL-2, IL-15, or IL-1β (11, 32). Similarly, NKp44 can be upregulated on plasmacytoid DCs upon culture with IL-3 (33). Therefore, NKp44 may also be considered a marker of cytokine-activated NK cells in humans. IL-2-induced upregulation of NKp44 on NK cells can be inhibited by prostaglandin E2, which is readily produced by tumor-associated fibroblasts, especially when exposed to NK cells in culture (34). Similarly, prednisolone can suppress IL-2-mediated upregulation of NKp44 (26).

The extracellular domains of NCRs consist of one (NKp30 and NKp44) or two (NKp46) Ig-like domains that are responsible for ligand binding (10, 12, 13). Ligand binding and signaling function by NKp30 is highly dependent upon integrity of the membrane proximal stalk region (35). Crystal structures have revealed that NKp30 and NKp44 can form homodimeric structures with NKp30 dimerizing in a head-to-tail fashion to form an I-type Ig-like fold and two NKp44 V-type Ig-like domains form a saddle-shaped dimer with unique disulfide bridging (36, 37). On the other hand, the crystal structure of NKp46 demonstrates two C2-type Ig-like domains that are folded and oriented similar to the Ig-like domains of KIRs (38). Evidence for a homodimerization interface within the membrane proximal Ig-like domain of NKp46 has also been reported, and disruption of this dimerization interaction prevented ligand binding and activating function of the receptor (39). In conclusion, the three NCRs have unrelated structures, and grouping these receptors together is based more on their shared functional properties than related structure or genetic evolution (13).

The transmembrane domains of all three NCRs contain a positively charged lysine (NKp44) or arginine (NKp30 and NKp46) residue that interacts with acidic aspartic acid residues found in the transmembrane regions of the adaptor proteins DAP12 (NKp44) or TCR-ζ and/or FcεRI-γ (NKp30 and NKp46) (10). Physical association with DAP12 *via* these transmembrane charged residues is essential for surface expression of NKp44 (40). The reductions in surface expression levels of NKp30 and NKp46 on “adaptive” or “memory-like” NK cells is associated with the lack of FcεRI-γ expression in these cells (29, 30), exemplifying the importance of associating with this specific adaptor to transport a functional receptor to the cell surface. In addition to promoting surface expression, physical association with these associated transmembrane adaptors provides potent activation signaling function to the NCRs, since the tyrosine phosphorylation of their cytoplasmic ITAM domains results in the recruitment and activation of the Syk and ZAP-70 protein tyrosine kinases (2, 41). A unique activation signaling crosstalk has been reported between the NCRs, in which engagement of one NCR appears to initiate signaling through the others (41). Curiously, while several mRNA splice variants encoding NKp44 have been described, the major protein product or isoform was found to also contain a cytoplasmic ITIM-like domain. Although early work suggested that this domain was incapable of providing inhibitory signaling function in an NK-like cell line (40), more recent work has demonstrated ITIM-mediated inhibitory function by NKp44 upon recognition of a specific ligand, proliferating cell nuclear antigen (PCNA), as detailed below (42).

## Ligands of the NCRs

**Figure 1:**
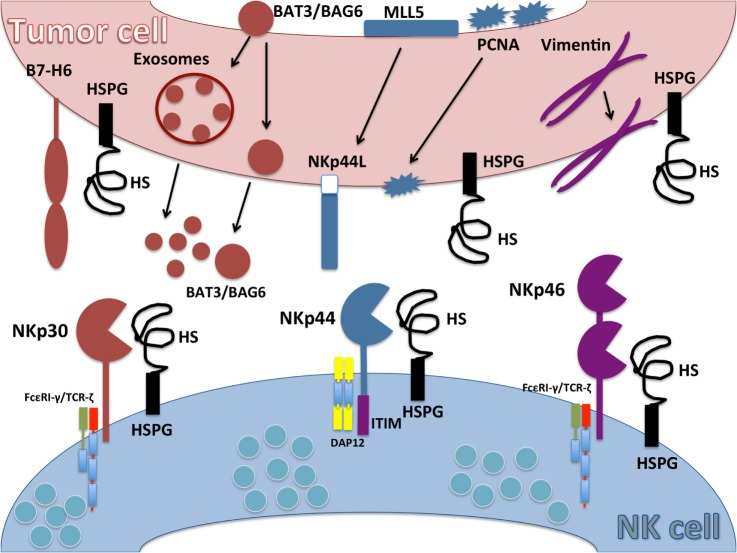
**Ligands for natural cytotoxicity receptors (NCRs)**. Schematic representation of NCR ligands on tumor cell and their interaction with NKp30, NKp44, and NKp46 on natural killer (NK) cells. B7-H6 is an activating ligand for NKp30 upregulated on tumor cells and absent on normal cells. HLA-B-associated transcript 3 (BAT3)/Bcl2-associated anthogene 6 (BAG6) expressed in the nucleus moves to the plasma cell membrane or is released in exosomes. NKp44L is a splice variant isoform of the nuclear protein Mixed-lineage leukemia-5 protein that localizes to the tumor cell plasma membrane to serve as an activating ligand for NKp44. Proliferating cell nuclear antigen (PCNA) is a nuclear protein involved in DNA replication and repair mechanisms that relocalizes to the plasma membrane to serve as an NKp44 inhibitory ligand. Cytoskeleton type III filamentous vimentin is an intracellular protein but can be upregulated on the cell surface of infected cells, where it serves as a ligand for NKp46. Heparan sulfate proteoglycans (HSPGs) can interact with all NCRs. Heparan sulfate (HS) expressed on NK cell surface (cis interaction) can mask interactions with HSPG or other ligands on target cells (trans interactions).

Despite a great deal of work by numerous research groups, our understanding of the ligands for NCRs is still not clearly established. A diverse array of molecules have been report to interact with the extracellular domains of NCRs, including carbohydrate-based contacts, cell surface proteins, and surprisingly, several intracellular-localized proteins that appear to reach the surface of infected or transformed cells. While engagement with most of these reported ligands stimulates activation of NK cells, some have been found to inhibit their functions. Our current understanding of putative ligands for NCRs and their functions are described below and summarized in Figure 1 and Table 1.

**Table 1:** **Ligands for natural cytotoxicity receptors**.

Receptor	Ligands	Function	Reference
NKp30	Hemagglutinin (HA) of human vaccinia virus	Inhibition	(43)
	pp65, Main tegument protein of human cytomegalovirus	Inhibition	(44)
	(DBL)-1α domain of *Plasmodium falciparum* erythrocyte membrane protein-1	Activation	(45)
	Heparan sulfate (HS) glycosaminoglycans (GAGs)	Activation/regulation	(46–48)
	BAT3/BAG6	Activation	(49–51)
	B7-H6	Activation	(52, 53)
NKp44	Redirected cytotoxicity and blockade of natural cytotoxicity with NKp44 antibody	Activation	(11)
	HA of influenza and Sendai viruses	Activation	(54, 55)
	HA from avian Newcastle disease	Activation	(56)
	Domain III of WNV envelope protein of West Nile and Dengue virus	Activation	(57)
	*Mycobacterium bovis bacillus* Calmette-Guérin (BCG)	Unclear	(58)
	Unknown ligand on cartilage-derived chondrocytes	Activation	(59)
	HS GAGs	Activation/regulation	(47, 60, 61)
	NKp44L	Activation	(62, 63)
	Proliferating cell nuclear antigen	Inhibition	(42, 64)
NKp46	HA of influenza virus	Activation	(65–69)
	HA of avian Newcastle disease	Activation	(56)
	HA of human vaccinia virus	Activation	(43)
	(DBL)-1α domain of *Plasmodium falciparum* erythrocyte membrane protein-1	Activation	(45)
	HS GAGs	Activation/regulation	(47)
	Vimentin	Activation	(70)

### Viral Ligands

Several viral-derived NCR ligands have been reported. Early work showed that the viral hemagglutinin (HA) of influenza virus on the surface of infected cells can readily engage with branched α-2,3- and α-2,6-sialylated *O*-glycan sequences conjugated on NKp46, and influenza-infected target cells can be killed by human NK cells in a NKp46-dependent manner (65–68). In fact, exposure of NK cells to influenza virions or free HA has been found to decrease NCR-mediated cytotoxicity, which was associated with loss of TCR-ζ protein expression (71). In addition, influenza virus-infected DCs stimulate IFN-γ production by NK cells in an NKp46- and HA-dependent manner (69). Furthermore, it has been demonstrated that NKp46-deficient mice are more susceptible to death after infection with influenza virus (72). These interactions are consistent with the known sialic acid-binding properties of viral HAs. HAs from influenza and Sendai viruses have further been shown to also interact with NKp44, but not NKp30, and NKp44^+^ NK cells can kill cells infected with these viruses (54, 55). Similarly, both NKp46 and NKp44 were found to interact with the HA from avian Newcastle disease virus and this interaction potentiates cytotoxicity of target cells infected with this paramyxovirus (56).

Hemagglutinin from the orthopox family viruses, human vaccinia virus, and murine ectromelia virus has been shown to interact with NKp46 and NKp30 (43). Late-stage vaccinia virus-infected target cells were further shown to be less susceptible to NK cell cytotoxicity compared to uninfected targets, and this reduced killing was dependent upon viral HA in the target cells and NKp30 in the NK cells (43). The results from this study suggest that the HA on the surface of vaccinia virus-infected cells interacts with NKp30 to either block its activating function or to mediate inhibitory signaling in NK cells, whereas NKp46 engagement with vaccinia virus-derived HA on target cell surfaces stimulates cytotoxicity responses.

NKp44 has been shown to recognize the envelope glycoproteins from West Nile and Dengue flaviviruses (57). In particular, NKp44 was found to directly bind domain III of WNV envelope protein, but does not appear to involve viral HA, since independent of sialylation of oligosaccharides on NKp44. Consistent with this finding, West Nile virus-infected cells more readily bind a soluble recombinant form of NKp44 and stimulate NK cells to degranulate and produce IFN-γ in an NKp44-dependent manner (57). It should be noted that expression of Dengue viral non-structural proteins in target cells reduces susceptibility to NK cell cytotoxicity through upregulating MHC-I expression (73).

In addition, NKp30 has been shown to directly interact with pp65, which is the main tegument protein of human cytomegalovirus (HCMV) (44). HCMV infected target cells were found to be less susceptible to NK cell-mediated killing, and this inhibition was lost if the target cells were infected with pp65-deficient HCMV or if anti-NKp30 blocking antibodies were added (44). The authors of this report further provided evidence that treating NK cells with a recombinant soluble form of pp65 resulted in the dissociation of the TCR-ζ signaling adaptor protein from NKp30 (44). In this way, pp65 appears to provide HCMV with a mechanism to avoid NK cell-mediated immunity by disrupting activation signaling through NKp30.

### Other Ligands Expressed by Pathogens or Pathological Conditions

Natural cytotoxicity receptors have also been shown to directly recognize bacterial and parasite pathogens. It has been demonstrated that NKp30 (and to a lesser extent, NKp46) can interact with the Duffy binding-like (DBL)-1α domain of *Plasmodium falciparum* erythrocyte membrane protein-1 to mediate cytolysis of malaria-infected erythrocytes (45). The interaction appears to be direct, since the effect can be inhibited by the addition of recombinant soluble forms of these NCRs or peptides matching the sequence of DBL-1α (45).

It has been reported that *Mycobacterium bovis bacillus* Calmette-Guérin (BCG) can directly interact with NKp44, and exposure to BCG can increase NKp44 expression on CD56^bright^ NK cells (58). This study also found that additional *Mycobacterium* family members can bind NKp44, such as *Nocardia farcinica* and *Pseudomonas aeruginosa*. Interactions of these bacteria with NKp44 did not activate NK cell functions, however, so the relevance of these interactions is currently unclear.

NKp44 has been reported to recognize an uncharacterized ligand on cartilage-derived chondrocytes, and cytotoxicity of primary chondrocytes by long-term IL-2 activated-NK cells was inhibited by an NKp44-blocking antibody (59). These results suggest that NKp44 activation signaling may promote NK cell-mediated autoimmunity in chronic inflammatory cartilaginous disease.

### Heparan Sulfates

Heparan sulfate (HS) glycosaminoglycans (GAGs) have also been shown to interact with all of the NCRs, with different affinities for the three receptors (46–48, 60). While these carbohydrate-directed interactions likely do not represent primary ligands for the NCRs, they appear to have the capacity to regulate NCR function or may play a supporting role as co-ligands (61). HS GAGs consist of long, unbranched, anionic polysaccharides that are found on cell surfaces and the extracellular matrix (74). The HS GAG polysaccharides are composed of repeating disaccharide units of uronic acid (iduronic or glucuronic acid) and glucosamine that are differentially sulfated at *N*, 2-*O*, 3-*O*, and 6-*O* positions to generate highly diverse structures with unique protein binding properties (75). HS GAG can be conjugated to a small subset of proteins to form HS proteoglycans, including syndecans and glypicans, and their negative-charged configurations can provide docking sites for basic domains on chemokines, FGF, and wnt ligand family members, thereby “presenting” them to cell surface receptors (76–79).

Interestingly, HS GAG are highly diverse structures, and the three NCRs preferentially recognize highly sulfated HS structures *via* basic amino acid patches on the receptor surfaces, and each NCR demonstrates distinct HS binding specificity (47, 48, 60). Therefore, it is conceivable that each NCR has the capacity to distinguish particular configurations of HS GAG primary and tertiary structures that might be uniquely expressed in the contexts of tumor microenvironments or sites of infection or inflammation. We have also shown the HS GAGs can interact with another NK cell receptor, KIR2DL4, and the binding can modulate receptor function (80). In addition, an interaction of NKp44 with the heparan sulfate proteoglycans (HSPGs), syndecan-4, in cis on the NK cell surface can modulate the surface distribution and function of the receptor (61). Based on this and other reports (47), we have proposed that interactions with HSPGs in cis (on the surface of NK cells) may be impacting KIR2DL4 and NCR functions through masking interactions with HS GAG or other ligands on adjacent target cells (trans interactions) and/or may be affecting the trafficking of NCR to intracellular degradation and recycling pathways upon endocytosis (61, 81). In this way, cis interactions between NCR and HSPGs may provide an allosteric regulation mechanism. It is also intriguing to speculate that treatment of patients with structurally related heparin as a therapeutic agent could impact NK cell functions through binding to NCRs and other NK cell surface receptors, including KIR2DL4.

### Intracellular Proteins As Cell Surface Ligands

The expression of a ligand for NKp44, named NKp44L, was first shown to be induced by the HIV-1 envelope protein gp41 on infected CD4^+^ T cells, and the expression increased in patients with increasing viral load (62). NKp44L is an activating ligand, since NK cell-mediated lysis of HIV-infected CD4^+^ T cells was inhibited by antibodies to NKp44 or NKp44L (62). The NKp44L was subsequently identified as a unique splice variant isoform of mixed-lineage leukemia-5 (MLL5) protein (63). While full-length MLL5 is a nuclear protein, the NKp44L splice variant is localized near the plasma membrane in the cytoplasm and expressed in several tumor tissues and transformed cell lines, but not in normal tissues (63).

It was reported that NKp46 is involved in NK cell-mediated cytolytic attack of monocytes infected with *Mycobacterium tuberculosis* (20). Subsequent work established that this is due to an interaction with vimentin, which is expressed at high levels in infected monocytes and appears on the cell surface (70). Since vimentin is a type III intermediate filament of the cytoskeleton, however, it is unexpected to find on the cell surface, but this follows an emerging theme of several traditionally intracellular proteins serving as putative cell surface ligands for NCRs. NK cells were more efficient at lysing target cells transfected to overexpress vimentin, and this cytotoxicity was inhibited by antibodies targeting NKp46 or vimentin (70).

In addition, NKp30 has been shown to interact with the HLA-B-associated transcript 3 (BAT3)/Bcl2-associated anthogene 6 (BAG6) protein to stimulate NK cytolytic responses (49). BAT3/BAG6 is predominantly expressed in the nucleus, but can move to the plasma membrane in cells exposed to heat shock and can be secreted in exosomes by tumors and stressed cells (49, 82). BAT3/BAG6-expressing exosomes can stimulate cytokine release from NK cells upon interaction with NKp30, and BAT3/BAG6 expression by DC is responsible for activation NK cells to mediate the crosstalk with DC (49, 50). Similarly, RIG-I stimulation of melanoma cell lines was shown to trigger the extracellular release of BAT3/BAG6-containing vesicles that can stimulate NK cell cytolytic responses (51). In contrast, a soluble form of BAT3/BAG6 has been found at high levels in the plasma of CLL patients and can suppress NK cytolytic responses, apparently by blocking recognition of this and other ligands on tumor cells (82, 83).

It was also reported that NKp44 can interact with PCNA, at target cell surfaces (42). PCNA is highly expressed in proliferating cancer cells, where it is usually tightly associated with DNA and involved in DNA replication and repair mechanisms (84). Surprisingly, PCNA was found to migrate to the plasma membrane of target cells within the immunological synapse with NKp44-expressing NK cells, and this interaction inhibited cytolytic function and IFN-γ production by the NK cells (42). A second report has also described the interaction of PCNA and NKp44 and the association of PCNA with MHC-I molecules at the plasma membrane of tumor cells as a potential surface transport mechanism (64). The PCNA-induced inhibition was found to be mediated through the ITIM-like sequence in the cytoplasmic domain of NKp44 (42), despite earlier work in which the ITIM-containing NKp44 cytoplasmic domain was shown to lack inhibitory function in the context of a chimeric receptor construct (40). It appears that PCNA interaction with the full NKp44 receptor establishes a unique conformation that transduces an inhibitory signal. Inhibitory function was also previously reported for NKp44 expressed on ILCs and plasmacytoid DCs (33, 85).

### B7-H6

NKp30 has been found to bind to a cell surface protein member of the B7 family, named B7-H6 (52). B7-H6 is not normally expressed on healthy cells, but can be upregulated on human tumor cells through a Myc-mediated mechanism (86) or upon stimulation of monocytes and neutrophils with TLR ligands or pro-inflammatory cytokines (87). Upon recognition by NKp30, B7-H6 triggers cytotoxicity and cytokine production by NK cells (52). The interaction of B7-H6 with NKp30 is the most rigorously characterized of NCR ligands, since it is the only NCR–ligand interaction so far confirmed in an X-ray crystallography structure (53). It has also been shown that some tumors can escape NKp30 recognition by shedding B7-H6 from their surfaces with the metalloproteases, ADAM-10, and ADAM-17 (88). Soluble and tumor-associated expression of B7-H6 in the peritoneum of ovarian cancer patients has also been shown to correlate with reduced surface expression of NKp30 on peritoneal NK cells, presumably due to chronic interaction with ligand (89).

## Splice Variants of NCRs Resulting in Distinct Receptor Isoforms

**Figure 2:**
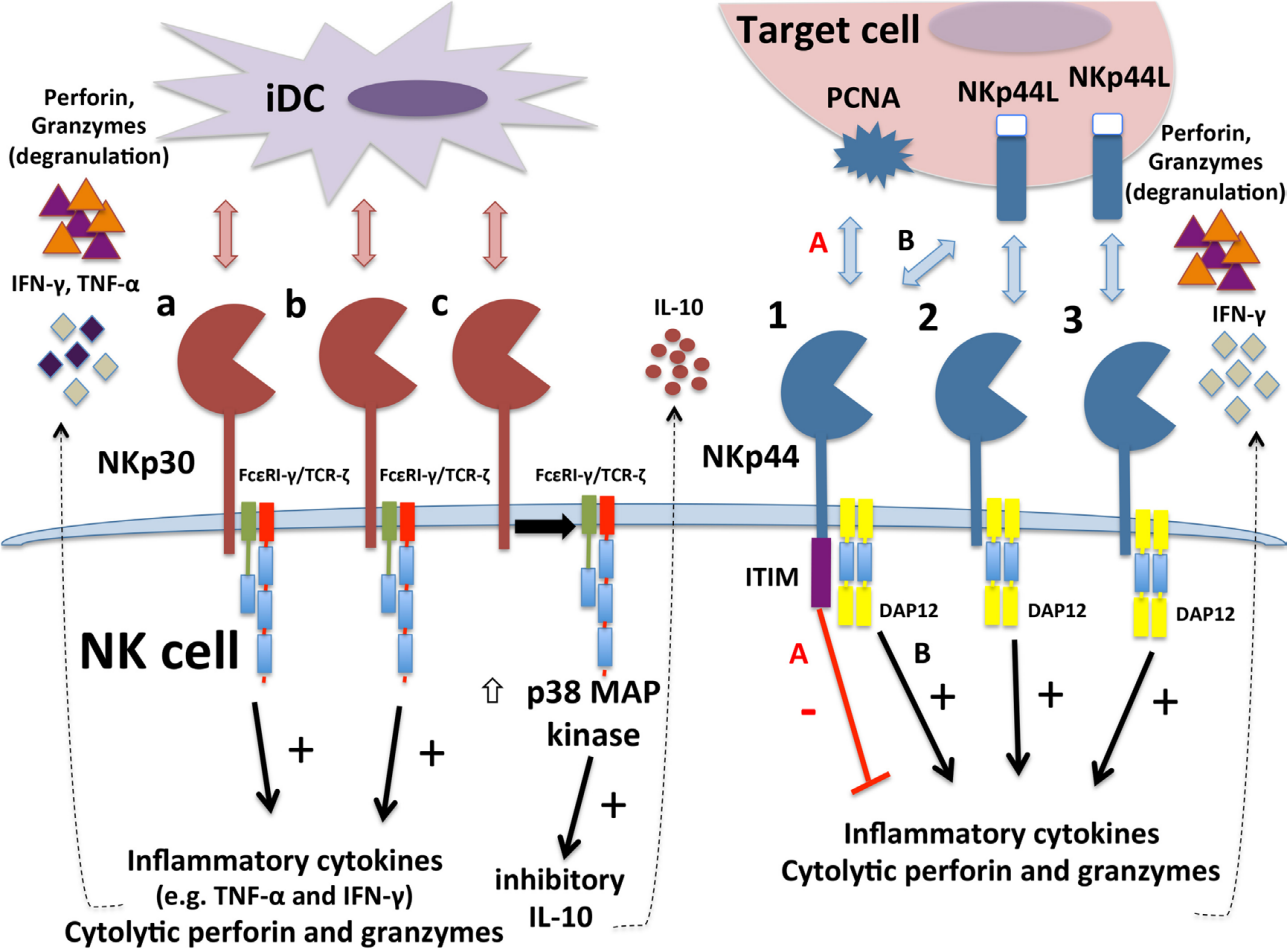
**Differential functions of different isoforms of NKp30 and NKp44**. Immunostimulatory NKp30a and NKp30b isoforms interaction with ligands on tumor target cells or immature DCs (iDCs) to stimulate degranulation responses and interferon (IFN)-γ production. Inhibitory NKp30c isoform instead produces inhibitory cytokine IL-10 upon engagement. NKp30c is also less tightly associated with TCR-ζ and triggers stronger activation of p38 MAP kinase. The NKp44-1 isoform contains a cytoplasmic ITIM that can transduce inhibitory signals when engaged with proliferating cell nuclear antigen (PCNA) ligand on target cell surface (pathway “A”), leading to decreased cytotoxicity and IFN-γ production. In contrast, all three NKp44 isoforms can engage with NKp44L to transduce activation signaling through DAP12 (pathway “B” for NKp44-1) that triggers cytotoxicity and IFN-γ production.

A variety of mRNA splice variants encoding different isoforms of NCRs have been recognized for many years, but only recent work has established that some of these variant NCR isoforms can facilitate inhibitory functions. Distinct splice variant expression patterns have also been shown to correlate with outcomes in cancer and infectious disease, suggesting potential prognostic value in patients. A summary diagram of current reported functions of distinct isoforms of NKp30 and NKp44 is presented in Figure 2.

### Splice Variants of *ncr3*

The expression of distinct isoforms of NKp30 is of great interest, because this NCR is involved in DC-to-NK cell crosstalk (25), can facilitate tumor cell recognition (13, 49, 90), and can influence the prognosis of different infectious diseases (91). Six splice variant transcripts have been identified from the *ncr3* gene, which was originally called 1C7 (92, 93). The most highly expressed *ncr3* variants are designated a, b, and c that encode NKp30 proteins with an extracellular V-type Ig domain, while d, e, and f isoforms encode NKp30 receptors possessing a C2-type Ig domain that lacks 25 amino acids (93). The three members of each subgroup share three distinct cytoplasmic domains encoded by splice variations within exon 4. Although the d, e, and f protein isoforms of NKp30 have not been studied to date, several groups have examined the functions and interesting clinical outcomes associated with differential expression of the a, b, and c isoforms, as discussed below.

Alternative splicing of the *ncr3* gene impacts functions of NKp30 isoforms that can be immunosuppressive or immunostimulatory. Delahaye and colleagues expressed isoforms NKp30a, NKp30b, and NKp30c in the human NK cell line NKL to characterize their functions. It was shown that antibody- or B7-H6-mediated engagement of NKp30 on NKL transfected with either NKp30a or NKp30b isoforms stimulated production of large amounts of IFN-γ, degranulation, and cytotoxicity responses. In contrast, engagement of NKp30c on NKL cell transfectants did not result in degranulation or elicit cytotoxicity, but instead produced the inhibitory cytokine IL-10 and very little IFN-γ (94). Similar immunostimulatory functions for NKp30a and NKp30b isoforms were observed when NKp30 transfectants were cocultured with DCs, whereas NKL-NKp30c transfectants demonstrated minimal responsiveness in these assays. In all of these experiments, NKp30a stimulated the most potent activating responses, whereas NKp30c was inhibitory or non-responsive, and NKp30b induced intermediate activation (94).

It was also shown that NKp30a associated more tightly with TCR-ζ upon crosslinking, as compared to NKp30c (94). Surprisingly, p38 MAP kinase activation was more pronounced when NKp30 was engaged in NKL-NKp30c cells then for NKL cells transfected with NKp30a or NKp30b. Furthermore, treatment of NKL-NKp30c cells with a p38 inhibitor produced IFN-γ upon exposure to immature DCs (94). Therefore, NKp30b and especially NKp30a are stimulatory isoforms that can induce cytotoxicity and cytokine production, whereas NKp30c induces an immunosuppressive response that appears to involve activation of p38 and the production of IL-10.

### NKp30 Isoforms and Cancer

In addition to characterizing different functions for distinct NKp30 isoforms, Delahaye performed a retrospective analysis of NKp30 expression profiles in 80 patients with gastrointestinal stromal tumors (GIST), which is a malignancy that expresses NKp30 ligands. In that analysis, predominant expression of the immunosuppressive NKp30c isoform over the immunostimulatory NKp30a/b isoforms was found to be associated with reduced overall survival in imatinib-treated patients (94). Moreover, a subset of GIST patients with predominant expression of the NKp30c isoform and a distinct haplotype involving two SNPs in the *ncr3* gene were found to be associated with particularly poor survival (94). For GI carcinomas and variety of other cancers, we observed that both cancerous and matched normal tissues manifested balanced NKp30c inhibitory and NKp30a/b activation profiles; yet, we found skewed NKp30 splice variant profiles in about 50% of a variety of tumor tissues compared to their matched normal tissues (95).

Neuroblastoma is another malignancy where NCRs are involved in the tumor cell recognition (96–98). Semeraro et al., found that neuroblastoma tumor samples express the NKp30 ligand, B7-H6, and some patients with metastatic neuroblastoma had high levels of soluble B7-H6 in their serum, which was associated with reduced expression of NKp30 on the surface of NK cells and higher degree of metastases (99). Furthermore, serum from patients with high soluble B7-H6 suppressed NK cell IFN-γ responses (99). Analysis of NKp30 isoform expression suggested lower expression of NKp30a/b isoforms in patients with metastatic neuroblastoma compared to patients with localized disease or healthy donors. Comparison of the ratios of expression of mRNA for individual NKp30 isoforms revealed that high expression of the suppressive NKp30c isoform over the activating NKp30b isoform was associated with shorter progression-free survival of patients with metastatic disease (99). Surprisingly, IFN-γ was found to suppress the expression of *B7-H6* and *BAT3* mRNA, whereas IL-10 increased *B7-H6* mRNA expression on neuroblastoma tumors. The results of this study suggest that high expression of IL-10 by NKp30c-expressing NK cells induces B7-H6 expression on tumor, thereby potentiating further expression of this immunosuppressive cytokine.

The potential prognostic value of NKp30 isoforms was also recently investigated in melanoma patients (100). The overall mRNA expression levels of each of the NKp30 isoforms were found to be reduced compared to expression in healthy donors, but expression ratios between the isoforms did not differ. Levels of mRNA expression of the immunosuppressive NKp30c isoform were found to be higher on NK cells from stage IV melanoma patients, but relative expression levels of each NKp30 isoform did not predict overall survival of patients. Interestingly, long-term surviving melanoma patients were found to express higher levels of stimulatory NKp30a transcript. In accordance with this finding, the long-term survivors were more likely to have SNPs associated with reduced expression of suppressive NKp30c and increased expression of NKp30a. Furthermore, NK cells from long-term surviving patients exhibited increased degranulation potential in response to NKp30 stimulation (100). Therefore, higher expression of NKp30a in melanoma patients appears to be beneficial.

### NKp30 Isoforms and Infectious Diseases

Surface expression NKp30 on NK cells was found to be downregulated in HIV-1 patients, but expression patterns of NKp30 isoforms do not affect disease progression or survival (101). NK cells from hepatitis C virus (HCV)-infected patients also showed reduced expression of NKp30 on the surface of NK cells, and expression of the immunosuppressive NKp30c transcript was found to be significantly decreased in infected patients compare to healthy controls (102). Also NKp30a/NKp30c ratio was significantly higher compared to healthy individuals suggesting an immunostimulatory profile in infected patients. If stratified according to mRNA expression levels, patients with low expression of all three isoforms had lower surface expression of NKp30 on NK cells, but higher ratio of NKp30a/NKp30c isoform transcripts. Accordingly, NK cells from these patients exhibited more potent degranulation and cytokine production responses upon engagement of the receptor, as compared to healthy controls (102). Interestingly, positive correlations were observed between NKp30a isoform mRNA levels and liver stiffness and between NKp30a/NKp30c ratio and a measure of liver fibrosis, suggesting that reduction of immunosuppressive NKp30c isoform and increase of immunostimulatory NKp30a isoform is associated with advanced liver disease in HCV-infected patients.

### NKp30 in the Decidua of Pregnant Women

Natural killer cells from decidual tissue in pregnant uterus (dNK) have unique phenotypical and functional properties compared to NK cells found in peripheral blood (pNK) (103). Mouse studies originally demonstrated that dNK cells function to support embryo implantation by secretion of numerous factors, including IFN-γ, to promote angiogenesis and trophoblast invasion (104). Comparison of freshly isolated pNK and dNK cells from the same pregnant donors showed that pNK cells express high levels of NKp30a and NKp30b isoforms and low levels of NKp30c isoform, while dNK cells had high levels of NKp30c but significantly lower amounts of NKp30a/b (105). While crosslinking NKp30 on IL-15-stimulated pNK cells induced degranulation, freshly isolated dNK cells did not degranulate, and co-crosslinking NKp30 in dNK cells did not inhibit NKp46-mediated degranulation, consistent with the lack of activation by NKp30c in previously described studies by Delahaye et al. (94, 105). Coculturing pNK cells in presence of IL-15 and IL-18 induced increased expression of NKp30a/b isoforms, whereas further addition of TGF-β suppressed the induction of expression of all three isoforms, although expression of NKp30b and NKp30c isoforms was greater than NKp30a upon addition of TGF-β (105). Overall, the combination of IL-15, IL-18, and TGF-β, which are found together in the decidual stromal microenvironment, shifted pNK cells toward higher expression of inhibitory NKp30c isoform and expression of other markers characteristic of dNK cells (105).

Shemesh et al. found significantly increased mRNA encoding the activating NKp30a/b isoforms in the placenta of women who had experienced sporadic or recurrent miscarriage within the first trimester, whereas this shift toward activating isoforms was not evident in the peripheral blood (106). The increase in activating isoforms did not correlate with higher expression of TNF-α, IFN-γ, IL-10, and placental growth factor mRNAs in the placental tissue of those women who experienced sporadic miscarriage, as compared to those that had undergone elective abortions. These results suggest that increased expression of these activating isoforms of NKp30 on dNK cells may be in some way contributing to failed pregnancies through promoting dysregulated cytokine production in the placenta.

### Splice Variants of *ncr2*

Three major mRNA splice variants of *ncr2* have been recognized, and one of these (NKp44-1) encodes the classic receptor that possesses a cytoplasmic ITIM, while the others (NKp44-2 and NKp44-3) have alternative sequences in the cytoplasmic region that lack ITIMs. It has long been known that IL-2-cultured NK cells upregulate expression of an activating form of NKp44-1 associated with the transmembrane signaling protein, DAP12, which becomes tyrosine phosphorylated upon receptor engagement with an antibody (11, 40, 41). Recent analysis of the major NKp44 isoforms, however, has demonstrated that isolated human NK cells cultured in IL-2- or IL-15 express predominantly NKp44-1 mRNA and have reduced capacity to kill PCNA-transfected target cells in an NKp44-dependent manner (12, 107). Furthermore, NK-92 cells transduced to overexpress NKp44-1 show suppressed cytotoxicity and diminished immune synapse formation toward PCNA-transfected target cells, as compared to NK-92 transduced to overexpress the other isotypes. These results indicate that the NKp44-1 isoform is an inhibitory receptor when engaged with the PCNA ligand expressed by target cells, whereas NKp44-2 and NKp44-3 do not transduce inhibitory signals (107).

### NKp44 Isoforms in Cancer

Shemesh et al. studied the impact of NKp44 isoforms on overall survival of patients with acute myeloid leukemia (AML) using TCGA RNA-Seq data (107). Whereas no survival advantage was found in newly diagnosed patients who expressed mRNA encoding NKp44, as compared to those lacking expression, survival was significantly diminished in patients who exclusively expressed NKp44-1, as compared to patients who also expressed at least some detectable level of NKp44-2 and/or NKp44-3 or lacked NKp44 expression altogether. These results imply that NKp44 plays a key role in NK cell responsiveness toward AML tumors, but exclusive expression of the ITIM-containing NKp44-1 isoform can stifle these beneficial responses, presumably through inhibitory signaling, thereby resulting in poor patient outcome.

The same group found higher incidence of NKp44 mRNA in various solid tumor tissues, as compared to surrounding normal tissues (95). Those tumor samples that expressed NKp44 mRNA were found to consist of predominantly NKp44-1 isoform. Thus, NK cells in the tumor microenvironment predominantly express the inhibitory NKp44 isoform.

### NKp44 in the Decidua of Pregnant Women

Siewiera et al. found that while NKp44-2 mRNA is expressed more by freshly isolated pNK cells than NKp44-1 and NKp44-3, dNK cells from women undergoing elective first trimester abortions (healthy pregnancies) express all three isoforms of NKp44 at similar levels (105). While crosslinking NKp44 on IL-15-stimulated pNK cells induces degranulation, crosslinking on freshly isolated dNK cells did not result in degranulation and co-crosslinking suppressed degranulation in response to crosslinking NKp46, demonstrating predominant inhibitory function for NKp44 in decidual/placental NK cells (105).

Shemesh et al. also found that NKp44-2 and NKp44-3 isoforms predominated in decidual tissue obtained from the majority of first trimester spontaneous abortions, while a NKp44-1-dominant (inhibitory) profile was found in dNK cells from most elective abortions or term deliveries (healthy pregnancies), which is consistent with the inhibitory function of dNK cells in the study by Siewiera et al. (95).

### Splice Variants of *ncr1*

Recent work by Shemer-Avni et al. has also provided new insights on differential splicing of *ncr1* (108). Five major splice variants of *ncr1* have been described, and three of these encode NKp46 protein isoforms containing both extracellular domains, while two lack the first Ig-like domain (D1, encoded by exon 3). Surprisingly, NK-92 cells transduced with a D1-negative *ncr1* cDNA degranulated significantly more efficiently toward HEK293T target cells, as compared to NK-92 cells transduced to express conventional NKp46 protein containing both Ig-like domains (108). Using NKp46-reactive antibodies, D1-negative NKp46 was not observed in fresh peripheral blood from healthy donors, but a subset expressing the NKp46 D1-negative receptor was found after long-time culturing of NK cells in IL-2 (108). This subset of NK cells containing D1-negative NKp46 degranulated more robustly in response to a combination of plate-bound anti-NKp46 and anti-NKp30 antibodies (108).

This group also studied NKp46 isoform expression in upper airway lavage samples from pediatric patients with respiratory tract viral infections. While most of these samples expressed both NKp30 and NKp46 mRNA, none contained mRNA encoding NKp44. When *ncr1* splice variants were analyzed, most of the lavage samples from virus-infected patients were found to contain D1-negative NKp46 isoform transcripts (108). Taken together, these results suggest that NK cells by IL-2 or viral infection can express isoforms of NKp46 lacking the D1 Ig-like domain, and NK cells expressing these domain-deficient receptors exhibit increased functional capacity.

## Conclusion

Significant progress has been made in recent years to improve our understanding of the functions and ligand recognition capacities of NCRs. Clearly these receptors play important roles in NK cell recognition of tissue changes in cancer, viral infections, decidual tissues in pregnancy, and immature DC. This new knowledge is crucial for establishing the basis of molecular mechanisms controlling NK cell responses under these diverse conditions. In contrast to the original dogma that NCRs are exclusively activating receptors, several new findings have revealed inhibitory functions for these receptors.

Numerous ligands or co-ligands for NCRs have now been described. Several of these can surprisingly trigger inhibitory signals or induce production of inhibitory cytokines when engaged with NCRs. Furthermore, some of these putative ligands are classically nuclear or cytosolic proteins that appear to relocate to the cell surface in cancer cells, where they can engage with NCRs. Also, heparan sulfates seem to have capacity to interact with all three of the NCRs and may regulate their functions in trans and cis.

The study of differentially spliced isoforms of NCRs has revealed surprising insights, since some of these isoforms elicit inhibitory function. Furthermore, dominant expression of the inhibitory forms has been linked to poor outcome in the context of cancer, but healthy outcome in pregnancy. Nonetheless, our understanding of the complexities of NCR isoforms is still in its infancy and requires a great deal of additional study.

Future work is clearly needed to sort out true NCR ligands and functional mechanisms responsible for the functions of some NCR isoforms, and their complexities are growing. Importantly, a firmer foundation of understanding promises to provide potential opportunities as prognostic indicators of disease status or opportunities to develop therapeutic strategies to manipulate NCRs on NK cells that could be beneficial to treat a wide variety of human pathologies.

## Author Contributions

TP and KC authored and edited the manuscript. AS, MB, and AP provided critical input and editing.

## Conflict of Interest Statement

The authors declare that the research was conducted in the absence of any commercial or financial relationships that could be construed as a potential conflict of interest.

## References

[1] Campbell KS Hasegawa J. Natural killer cell biology: an update and future directions. J Allergy Clin Immunol (2013) 132:536–44. 10.1016/j.jaci.2013.07.006 23906377 PMC3775709

[2] MacFarlane AW 4th Campbell KS. Signal transduction in natural killer cells. Curr Top Microbiol Immunol (2006) 298:23–57. 16329184 10.1007/3-540-27743-9_2

[3] Lanier LL. Up on the tightrope: natural killer cell activation and inhibition. Nat Immunol (2008) 9:495–502. 10.1038/ni1581 18425106 PMC2669298

[4] Vivier E Tomasello E Baratin M Walzer T Ugolini S. Functions of natural killer cells. Nat Immunol (2008) 9:503–10. 10.1038/ni1582 18425107

[5] Moretta A Bottino C Vitale M Pende D Cantoni C Mingari MC Activating receptors and coreceptors involved in human natural killer cell-mediated cytolysis. Annu Rev Immunol (2001) 19:197–223. 10.1146/annurev.immunol.19.1.197 11244035

[6] Kruse PH Matta J Ugolini S Vivier E. Natural cytotoxicity receptors and their ligands. Immunol Cell Biol (2014) 92:221–9. 10.1038/icb.2013.98 24366519

[7] Hudspeth K Silva-Santos B Mavilio D. Natural cytotoxicity receptors: broader expression patterns and functions in innate and adaptive immune cells. Front Immunol (2013) 4:69. 10.3389/fimmu.2013.00069 23518691 PMC3603285

[8] Diefenbach A Colonna M Koyasu S. Development, differentiation, and diversity of innate lymphoid cells. Immunity (2014) 41:354–65. 10.1016/j.immuni.2014.09.005 25238093 PMC4171710

[9] Sivori S Vitale M Morelli L Sanseverino L Augugliaro R Bottino C p46, a novel natural killer cell-specific surface molecule that mediates cell activation. J Exp Med (1997) 186:1129–36. 10.1084/jem.186.7.1129 9314561 PMC2211712

[10] Pessino A Sivori S Bottino C Malaspina A Morelli L Moretta L Molecular cloning of NKp46: a novel member of the immunoglobulin superfamily involved in triggering of natural cytotoxicity. J Exp Med (1998) 188:953–60. 10.1084/jem.188.5.953 9730896 PMC3207313

[11] Vitale M Bottino C Sivori S Sanseverino L Castriconi R Marcenaro E NKp44, a novel triggering surface molecule specifically expressed by activated natural killer cells, is involved in non-major histocompatibility complex-restricted tumor cell lysis. J Exp Med (1998) 187:2065–72. 10.1084/jem.187.12.2065 9625766 PMC2212362

[12] Cantoni C Bottino C Vitale M Pessino A Augugliaro R Malaspina A NKp44, a triggering receptor involved in tumor cell lysis by activating human natural killer cells, is a novel member of the immunoglobulin superfamily. J Exp Med (1999) 189:787–96. 10.1084/jem.189.5.787 10049942 PMC2192947

[13] Pende D Parolini S Pessino A Sivori S Augugliaro R Morelli L Identification and molecular characterization of NKp30, a novel triggering receptor involved in natural cytotoxicity mediated by human natural killer cells. J Exp Med (1999) 190:1505–16. 10.1084/jem.190.10.1505 10562324 PMC2195691

[14] Hollyoake M Campbell RD Aguado B. NKp30 (NCR3) is a pseudogene in 12 inbred and wild mouse strains, but an expressed gene in *Mus caroli*. Mol Biol Evol (2005) 22:1661–72. 10.1093/molbev/msi162 15872155

[15] Backman-Petersson E Miller JR Hollyoake M Aguado B Butcher GW. Molecular characterization of the novel rat NK receptor 1C7. Eur J Immunol (2003) 33:342–51. 10.1002/immu.200310008 12548565

[16] Hsieh CL Ogura Y Obara H Ali UA Rodriguez GM Nepomuceno RR Identification, cloning, and characterization of a novel rat natural killer receptor, RNKP30: a molecule expressed in liver allografts. Transplantation (2004) 77:121–8. 10.1097/01.TP.0000110423.27977.6F 14724446

[17] Walzer T Blery M Chaix J Fuseri N Chasson L Robbins SH Identification, activation, and selective in vivo ablation of mouse NK cells via NKp46. Proc Natl Acad Sci U S A (2007) 104:3384–9. 10.1073/pnas.0609692104 17360655 PMC1805551

[18] Tomasello E Yessaad N Gregoire E Hudspeth K Luci C Mavilio D Mapping of NKp46(+) cells in healthy human lymphoid and non-lymphoid tissues. Front Immunol (2012) 3:344. 10.3389/fimmu.2012.00344 23181063 PMC3501723

[19] Sivori S Pende D Bottino C Marcenaro E Pessino A Biassoni R NKp46 is the major triggering receptor involved in the natural cytotoxicity of fresh or cultured human NK cells. Correlation between surface density of NKp46 and natural cytotoxicity against autologous, allogeneic or xenogeneic target cells. Eur J Immunol (1999) 1999:1656–66. 10.1002/(SICI)1521-4141(199905)29:05<1656::AID-IMMU1656>3.0.CO;2-1 10359120

[20] Vankayalapati R Wizel B Weis SE Safi H Lakey DL Mandelboim O The NKp46 receptor contributes to NK cell lysis of mononuclear phagocytes infected with an intracellular bacterium. J Immunol (2002) 168:3451–7. 10.4049/jimmunol.168.7.3451 11907104

[21] Gur C Porgador A Elboim M Gazit R Mizrahi S Stern-Ginossar N The activating receptor NKp46 is essential for the development of type 1 diabetes. Nat Immunol (2010) 11:121–8. 10.1038/ni.1834 20023661

[22] Cagnano E Hershkovitz O Zilka A Bar-Ilan A Golder A Sion-Vardy N Expression of ligands to NKp46 in benign and malignant melanocytes. J Invest Dermatol (2007) 128:972–9. 10.1038/sj/jid.5701111 17972960

[23] Della Chiesa M Carlomagno S Frumento G Balsamo M Cantoni C Conte R The tryptophan catabolite l-kynurenine inhibits the surface expression of NKp46- and NKG2D-activating receptors and regulates NK-cell function. Blood (2006) 108:4118–25. 10.1182/blood-2006-03-006700 16902152

[24] Ferlazzo G Tsang ML Moretta L Melioli G Steinman RM Munz C. Human dendritic cells activate resting natural killer (NK) cells and are recognized via the NKp30 receptor by activated NK cells. J Exp Med (2002) 195:343–51. 10.1084/jem.20011149 11828009 PMC2193591

[25] Vitale M Della Chiesa M Carlomagno S Pende D Arico M Moretta L NK-dependent DC maturation is mediated by TNFalpha and IFNgamma released upon engagement of the NKp30 triggering receptor. Blood (2005) 106:566–71. 10.1182/blood-2004-10-4035 15784725

[26] Vitale C Chiossone L Cantoni C Morreale G Cottalasso F Moretti S The corticosteroid-induced inhibitory effect on NK cell function reflects down-regulation and/or dysfunction of triggering receptors involved in natural cytotoxicity. Eur J Immunol (2004) 34:3028–38. 10.1002/eji.200425418 15368269

[27] Mavoungou E Bouyou-Akotet MK Kremsner PG Effects of prolactin and cortisol on natural killer (NK) cell surface expression and function of human natural cytotoxicity receptors (NKp46, NKp44 and NKp30). Clin Exp Immunol (2005) 139:287–96. 10.1111/j.1365-2249.2004.02686.x 15654827 PMC1809301

[28] Bozzano F Picciotto A Costa P Marras F Fazio V Hirsch I Activating NK cell receptor expression/function (NKp30, NKp46, DNAM-1) during chronic viraemic HCV infection is associated with the outcome of combined treatment. Eur J Immunol (2011) 41:2905–14. 10.1002/eji.201041361 21695691

[29] Lee J Zhang T Hwang I Kim A Nitschke L Kim M Epigenetic modification and antibody-dependent expansion of memory-like NK cells in human cytomegalovirus-infected individuals. Immunity (2015) 42:431–42. 10.1016/j.immuni.2015.02.013 25786175 PMC4537797

[30] Schlums H Cichocki F Tesi B Theorell J Beziat V Holmes TD Cytomegalovirus infection drives adaptive epigenetic diversification of NK cells with altered signaling and effector function. Immunity (2015) 42:443–56. 10.1016/j.immuni.2015.02.008 25786176 PMC4612277

[31] Castriconi R Cantoni C Della Chiesa M Vitale M Marcenaro E Conte R Transforming growth factor beta 1 inhibits expression of NKp30 and NKG2D receptors: consequences for the NK-mediated killing of dendritic cells. Proc Natl Acad Sci U S A (2003) 100:4120–5. 10.1073/pnas.0730640100 12646700 PMC153058

[32] Mattiola I Pesant M Tentorio PF Molgora M Marcenaro E Lugli E Priming of human resting NK cells by autologous M1 macrophages via the engagement of IL-1beta, IFN-beta, and IL-15 pathways. J Immunol (2015) 195:2818–28. 10.4049/jimmunol.1500325 26276870

[33] Bonaccorsi I Cantoni C Carrega P Oliveri D Lui G Conte R The immune inhibitory receptor LAIR-1 is highly expressed by plasmacytoid dendritic cells and acts complementary with NKp44 to control IFNalpha production. PLoS One (2010) 5:e15080 10.1371/journal.pone.0015080 21151495 PMC2994815

[34] Balsamo M Scordamaglia F Pietra G Manzini C Cantoni C Boitano M Melanoma-associated fibroblasts modulate NK cell phenotype and antitumor cytotoxicity. Proc Natl Acad Sci U S A (2009) 106:20847–52. 10.1073/pnas.0906481106 19934056 PMC2791633

[35] Memmer S Weil S Beyer S Zoller T Peters E Hartmann J The stalk domain of NKp30 contributes to ligand binding and signaling of a preassembled NKp30-CD3zeta complex. J Biol Chem (2016) 291:25427–38. 10.1074/jbc.M116.742981 27754869 PMC5207244

[36] Cantoni C Ponassi M Biassoni R Conte R Spallarossa A Moretta A The three-dimensional structure of the human NK cell receptor NKp44, a triggering partner in natural cytotoxicity. Structure (2003) 11:725–34. 10.1016/S0969-2126(03)00095-9 12791260

[37] Joyce MG Tran P Zhuravleva MA Jaw J Colonna M Sun PD. Crystal structure of human natural cytotoxicity receptor NKp30 and identification of its ligand binding site. Proc Natl Acad Sci U S A (2011) 108:6223–8. 10.1073/pnas.1100622108 21444796 PMC3076882

[38] Foster CE Colonna M Sun PD. Crystal structure of the human natural killer (NK) cell activating receptor NKp46 reveals structural relationship to other leukocyte receptor complex immunoreceptors. J Biol Chem (2003) 278:46081–6. 10.1074/jbc.M308491200 12960161

[39] Jaron-Mendelson M Yossef R Appel MY Zilka A Hadad U Afergan F Dimerization of NKp46 receptor is essential for NKp46-mediated lysis: characterization of the dimerization site by epitope mapping. J Immunol (2012) 188:6165–74. 10.4049/jimmunol.1102496 22615207

[40] Campbell KS Yusa S Kikuchi-Maki A Catina TL. NKp44 triggers NK cell activation through DAP12 association that is not influenced by a putative cytoplasmic inhibitory sequence. J Immunol (2004) 172:899–906. 10.4049/jimmunol.172.2.899 14707061

[41] Augugliaro R Parolini S Castriconi R Marcenaro E Cantoni C Nanni M Selective cross-talk among natural cytotoxicity receptors in human natural killer cells. Eur J Immunol (2003) 33:1235–41. 10.1002/eji.200323896 12731048

[42] Rosental B Brusilovsky M Hadad U Oz D Appel MY Afergan F Proliferating cell nuclear antigen is a novel inhibitory ligand for the natural cytotoxicity receptor NKp44. J Immunol (2011) 187:5693–702. 10.4049/jimmunol.1102267 22021614 PMC3269963

[43] Jarahian M Fiedler M Cohnen A Djandji D Hammerling GJ Gati C Modulation of NKp30- and NKp46-mediated natural killer cell responses by poxviral hemagglutinin. PLoS Pathog (2011) 7:e1002195. 10.1371/journal.ppat.1002195 21901096 PMC3161980

[44] Arnon TI Achdout H Levi O Markel G Saleh N Katz G Inhibition of the NKp30 activating receptor by pp65 of human cytomegalovirus. Nat Immunol (2005) 6:515–23. 10.1038/ni1190 15821739

[45] Mavoungou E Held J Mewono L Kremsner PG. A Duffy binding-like domain is involved in the NKp30-mediated recognition of *Plasmodium falciparum*-parasitized erythrocytes by natural killer cells. J Infect Dis (2007) 195:1521–31. 10.1086/515579 17436233

[46] Bloushtain N Qimron U Bar-Ilan A Hershkovitz O Gazit R Fima E Membrane-associated heparan sulfate proteoglycans are involved in the recognition of cellular targets by NKp30 and NKp46. J Immunol (2004) 173:2392–401. 10.4049/jimmunol.173.4.2392 15294952

[47] Hecht ML Rosental B Horlacher T Hershkovitz O De Paz JL Noti C Natural cytotoxicity receptors NKp30, NKp44 and NKp46 bind to different heparan sulfate/heparin sequences. J Proteome Res (2009) 8:712–20. 10.1021/pr800747c 19196184

[48] Hershkovitz O Jarahian M Zilka A Bar-Ilan A Landau G Jivov S Altered glycosylation of recombinant NKp30 hampers binding to heparan sulfate: a lesson for the use of recombinant immuno-receptors as an immunological tool. Glycobiology (2008) 18:28–41. 10.1093/glycob/cwm125 18006589

[49] Pogge von Strandmann E Simhadri VR Von Tresckow B Sasse S Reiners KS Hansen HP Human leukocyte antigen-B-associated transcript 3 is released from tumor cells and engages the NKp30 receptor on natural killer cells. Immunity (2007) 27:965–74. 10.1016/j.immuni.2007.10.010 18055229

[50] Simhadri VR Reiners KS Hansen HP Topolar D Simhadri VL Nohroudi K Dendritic cells release HLA-B-associated transcript-3 positive exosomes to regulate natural killer function. PLoS One (2008) 3:e3377. 10.1371/journal.pone.0003377 18852879 PMC2566590

[51] Dassler-Plenker J Reiners KS Van Den Boorn JG Hansen HP Putschli B Barnert S RIG-I activation induces the release of extracellular vesicles with antitumor activity. Oncoimmunology (2016) 5:e1219827. 10.1080/2162402X.2016.1219827 27853642 PMC5087302

[52] Brandt CS Baratin M Yi EC Kennedy J Gao Z Fox B The B7 family member B7-H6 is a tumor cell ligand for the activating natural killer cell receptor NKp30 in humans. J Exp Med (2009) 206:1495–503. 10.1084/jem.20090681 19528259 PMC2715080

[53] Xu X Narni-Mancinelli E Cantoni C Li Y Guia S Gauthier L Structural insights into the inhibitory mechanism of an antibody against B7-H6, a stress-induced cellular ligand for the natural killer cell receptor NKp30. J Mol Biol (2016) 428:4457–66. 10.1016/j.jmb.2016.09.011 27663271

[54] Arnon TI Lev M Katz G Chernobrov Y Porgador A Mandelboim O. Recognition of viral hemagglutinins by NKp44 but not by NKp30. Eur J Immunol (2001) 31:2680–9. 10.1002/1521-4141(200109)31:9<2680::AID-IMMU2680>3.0.CO;2-A 11536166

[55] Ho JW Hershkovitz O Peiris M Zilka A Bar-Ilan A Nal B H5-type influenza virus hemagglutinin is functionally recognized by the natural killer-activating receptor NKp44. J Virol (2008) 82:2028–32. 10.1128/JVI.02065-07 18077718 PMC2258730

[56] Jarahian M Watzl C Fournier P Arnold A Djandji D Zahedi S Activation of natural killer cells by Newcastle disease virus hemagglutinin-neuraminidase. J Virol (2009) 83:8108–21. 10.1128/JVI.00211-09 19515783 PMC2715740

[57] Hershkovitz O Rosental B Rosenberg LA Navarro-Sanchez ME Jivov S Zilka A NKp44 receptor mediates interaction of the envelope glycoproteins from the West Nile and dengue viruses with NK cells. J Immunol (2009) 183:2610–21. 10.4049/jimmunol.0802806 19635919 PMC2768489

[58] Esin S Batoni G Counoupas C Stringaro A Brancatisano FL Colone M Direct binding of human NK cell natural cytotoxicity receptor NKp44 to the surfaces of mycobacteria and other bacteria. Infect Immun (2008) 76:1719–27. 10.1128/IAI.00870-07 18212080 PMC2292874

[59] Bialoszewska A Baychelier F Niderla-Bielinska J Czop A Debre P Vieillard V Constitutive expression of ligand for natural killer cell NKp44 receptor (NKp44L) by normal human articular chondrocytes. Cell Immunol (2013) 285:6–9. 10.1016/j.cellimm.2013.08.005 24044960

[60] Hershkovitz O Jivov S Bloushtain N Zilka A Landau G Bar-Ilan A Characterization of the recognition of tumor cells by the natural cytotoxicity receptor, NKp44. Biochemistry (2007) 46:7426–36. 10.1021/bi7000455 17536787

[61] Brusilovsky M Radinsky O Cohen L Yossef R Shemesh A Braiman A Regulation of natural cytotoxicity receptors by heparan sulfate proteoglycans in -cis: a lesson from NKp44. Eur J Immunol (2015) 45:1180–91. 10.1002/eji.201445177 25546090 PMC4415513

[62] Vieillard V Strominger JL Debre P. NK cytotoxicity against CD4+ T cells during HIV-1 infection: a gp41 peptide induces the expression of an NKp44 ligand. Proc Natl Acad Sci U S A (2005) 102:10981–6. 10.1073/pnas.0504315102 16046540 PMC1180624

[63] Baychelier F Sennepin A Ermonval M Dorgham K Debre P Vieillard V. Identification of a cellular ligand for the natural cytotoxicity receptor NKp44. Blood (2013) 122:2935–42. 10.1182/blood-2013-03-489054 23958951

[64] Horton NC Mathew SO Mathew PA. Novel interaction between proliferating cell nuclear antigen and HLA I on the surface of tumor cells inhibits NK cell function through NKp44. PLoS One (2013) 8:e59552. 10.1371/journal.pone.0059552 23527218 PMC3602199

[65] Mandelboim O Lieberman N Lev M Paul L Arnon TI Bushkin Y Recognition of haemagglutinins on virus-infected cells by NKp46 activates lysis by human NK cells. Nature (2001) 409:1055–60. 10.1038/35059110 11234016

[66] Arnon TI Achdout H Lieberman N Gazit R Gonen-Gross T Katz G The mechanisms controlling the recognition of tumor- and virus-infected cells by NKp46. Blood (2004) 103:664–72. 10.1182/blood-2003-05-1716 14504081

[67] Achdout H Meningher T Hirsh S Glasner A Bar-On Y Gur C Killing of avian and swine influenza virus by natural killer cells. J Virol (2010) 84:3993–4001. 10.1128/JVI.02289-09 20130050 PMC2849486

[68] Mendelson M Tekoah Y Zilka A Gershoni-Yahalom O Gazit R Achdout H NKp46 O-glycan sequences that are involved in the interaction with hemagglutinin type 1 of influenza virus. J Virol (2010) 84:3789–97. 10.1128/JVI.01815-09 20147410 PMC2849520

[69] Draghi M Pashine A Sanjanwala B Gendzekhadze K Cantoni C Cosman D NKp46 and NKG2D recognition of infected dendritic cells is necessary for NK cell activation in the human response to influenza infection. J Immunol (2007) 178:2688–98. 10.4049/jimmunol.178.5.2688 17312110

[70] Garg A Barnes PF Porgador A Roy S Wu S Nanda JS Vimentin expressed on *Mycobacterium tuberculosis*-infected human monocytes is involved in binding to the NKp46 receptor. J Immunol (2006) 177:6192–8. 10.4049/jimmunol.177.9.6192 17056548

[71] Mao H Tu W Liu Y Qin G Zheng J Chan PL Inhibition of human natural killer cell activity by influenza virions and hemagglutinin. J Virol (2010) 84:4148–57. 10.1128/JVI.02340-09 20164232 PMC2863726

[72] Gazit R Gruda R Elboim M Arnon TI Katz G Achdout H Lethal influenza infection in the absence of the natural killer cell receptor gene Ncr1. Nat Immunol (2006) 7:517–23. 10.1038/ni1322 16565719

[73] Hershkovitz O Zilka A Bar-Ilan A Abutbul S Davidson A Mazzon M Dengue virus replicon expressing the nonstructural proteins suffices to enhance membrane expression of HLA class I and inhibit lysis by human NK cells. J Virol (2008) 82:7666–76. 10.1128/JVI.02274-07 18508882 PMC2493327

[74] Nakato H Kimata K. Heparan sulfate fine structure and specificity of proteoglycan functions. Biochim Biophys Acta (2002) 1573:312–8. 10.1016/S0304-4165(02)00398-7 12417413

[75] Sasisekharan R Venkataraman G. Heparin and heparan sulfate: biosynthesis, structure and function. Curr Opin Chem Biol (2000) 4:626–31. 10.1016/S1367-5931(00)00145-9 11102866

[76] Baeg GH Lin X Khare N Baumgartner S Perrimon N. Heparan sulfate proteoglycans are critical for the organization of the extracellular distribution of Wingless. Development (2001) 128:87–94. 11092814 10.1242/dev.128.1.87

[77] Loo BM Salmivirta M. Heparin/heparan sulfate domains in binding and signaling of fibroblast growth factor 8b. J Biol Chem (2002) 277:32616–23. 10.1074/jbc.M204961200 12077148

[78] Laguri C Arenzana-Seisdedos F Lortat-Jacob H. Relationships between glycosaminoglycan and receptor binding sites in chemokines – the CXCL12 example. Carbohydr Res (2008) 343:2018–23. 10.1016/j.carres.2008.01.047 18334249

[79] Lortat-Jacob H. The molecular basis and functional implications of chemokine interactions with heparan sulphate. Curr Opin Struct Biol (2009) 19:543–8. 10.1016/j.sbi.2009.09.003 19800217

[80] Brusilovsky M Cordoba M Rosental B Hershkovitz O Andrake MD Pecherskaya A Genome-wide siRNA screen reveals a new cellular partner of NK cell receptor KIR2DL4: heparan sulfate directly modulates KIR2DL4-mediated responses. J Immunol (2013) 191:5256–67. 10.4049/jimmunol.1302079 24127555 PMC3836631

[81] Brusilovsky M Radinsky O Yossef R Campbell KS Porgador A Carbohydrate-mediated modulation of NK cell receptor function: structural and functional influences of heparan sulfate moieties expressed on NK cell surface. Front Oncol (2014) 4:185 10.3389/fonc.2014.00185 25077071 PMC4100077

[82] Reiners KS Topolar D Henke A Simhadri VR Kessler J Sauer M Soluble ligands for NK cell receptors promote evasion of chronic lymphocytic leukemia cells from NK cell anti-tumor activity. Blood (2013) 121:3658–65. 10.1182/blood-2013-01-476606 23509156 PMC3643764

[83] Binici J Hartmann J Herrmann J Schreiber C Beyer S Guler G A soluble fragment of the tumor antigen BCL2-associated athanogene 6 (BAG-6) is essential and sufficient for inhibition of NKp30 receptor-dependent cytotoxicity of natural killer cells. J Biol Chem (2013) 288:34295–303. 10.1074/jbc.M113.483602 24133212 PMC3843045

[84] Stoimenov I Helleday T PCNA on the crossroad of cancer. Biochem Soc Trans (2009) 37:605–13. 10.1042/BST0370605 19442257

[85] Fuchs A Cella M Kondo T Colonna M. Paradoxic inhibition of human natural interferon-producing cells by the activating receptor NKp44. Blood (2005) 106:2076–82. 10.1182/blood-2004-12-4802 15941912

[86] Textor S Bossler F Henrich KO Gartlgruber M Pollmann J Fiegler N The proto-oncogene Myc drives expression of the NK cell-activating NKp30 ligand B7-H6 in tumor cells. Oncoimmunology (2016) 5:e1116674. 10.1080/2162402X.2015.1116674 27622013 PMC5007025

[87] Matta J Baratin M Chiche L Forel JM Cognet C Thomas G Induction of B7-H6, a ligand for the natural killer cell-activating receptor NKp30, in inflammatory conditions. Blood (2013) 122:394–404. 10.1182/blood-2013-01-481705 23687088

[88] Schlecker E Fiegler N Arnold A Altevogt P Rose-John S Moldenhauer G Metalloprotease-mediated tumor cell shedding of B7-H6, the ligand of the natural killer cell-activating receptor NKp30. Cancer Res (2014) 74:3429–40. 10.1158/0008-5472.CAN-13-3017 24780758

[89] Pesce S Tabellini G Cantoni C Patrizi O Coltrini D Rampinelli F B7-H6-mediated downregulation of NKp30 in NK cells contributes to ovarian carcinoma immune escape. Oncoimmunology (2015) 4:e1001224. 10.1080/2162402X.2014.1001224 26137398 PMC4485754

[90] Fiegler N Textor S Arnold A Rolle A Oehme I Breuhahn K Downregulation of the activating NKp30 ligand B7-H6 by HDAC inhibitors impairs tumor cell recognition by NK cells. Blood (2013) 122:684–93. 10.1182/blood-2013-02-482513 23801635

[91] Marras F Bozzano F Ascierto ML De Maria A. Baseline and dynamic expression of activating NK cell receptors in the control of chronic viral infections: the paradigm of HIV-1 and HCV. Front Immunol (2014) 5:305. 10.3389/fimmu.2014.00305 25071766 PMC4078246

[92] Nalabolu SR Shukla H Nallur G Parimoo S Weissman SM. Genes in a 220-kb region spanning the TNF cluster in human MHC. Genomics (1996) 31:215–22. 10.1006/geno.1996.0034 8824804

[93] Neville MJ Campbell RD. A new member of the Ig superfamily and a V-ATPase G subunit are among the predicted products of novel genes close to the TNF locus in the human MHC. J Immunol (1999) 162:4745–54. 10202016

[94] Delahaye NF Rusakiewicz S Martins I Menard C Roux S Lyonnet L Alternatively spliced NKp30 isoforms affect the prognosis of gastrointestinal stromal tumors. Nat Med (2011) 17:700–7. 10.1038/nm.2366 21552268

[95] Shemesh A Kugel A Steiner N Yezersky M Tirosh D Edri A NKp44 and NKp30 splice variant profiles in decidua and tumor tissues: a comparative viewpoint. Oncotarget (2016) 7:70912–23. 10.18632/oncotarget.12292 27765926 PMC5342598

[96] Bottino C Falco M Sivori S Moretta L Moretta A Biassoni R. Identification and molecular characterization of a natural mutant of the p50.2/KIR2DS2 activating NK receptor that fails to mediate NK cell triggering. Eur J Immunol (2000) 30:3569–74. 10.1002/1521-4141(200012)30:12<3569::AID-IMMU3569>3.0.CO;2-E 11169398

[97] Sivori S Parolini S Marcenaro E Castriconi R Pende D Millo R Involvement of natural cytotoxicity receptors in human natural killer cell-mediated lysis of neuroblastoma and glioblastoma cell lines. J Neuroimmunol (2000) 107:220–5. 10.1016/S0165-5728(00)00221-6 10854660

[98] Bottino C Dondero A Bellora F Moretta L Locatelli F Pistoia V Natural killer cells and neuroblastoma: tumor recognition, escape mechanisms, and possible novel immunotherapeutic approaches. Front Immunol (2014) 5:56. 10.3389/fimmu.2014.00056 24575100 PMC3921882

[99] Semeraro M Rusakiewicz S Minard-Colin V Delahaye NF Enot D Vely F Clinical impact of the NKp30/B7-H6 axis in high-risk neuroblastoma patients. Sci Transl Med (2015) 7:283ra255. 10.1126/scitranslmed.aaa2327 25877893

[100] Messaoudene M Fregni G Enot D Jacquelot N Neves E Germaud N NKp30 isoforms and NKp46 transcripts in metastatic melanoma patients: unique NKp30 pattern in rare melanoma patients with favorable evolution. Oncoimmunology (2016) 5:e1154251. 10.1080/2162402X.2016.1154251 28123867 PMC5214533

[101] Prada N Antoni G Commo F Rusakiewicz S Semeraro M Boufassa F Analysis of NKp30/NCR3 isoforms in untreated HIV-1-infected patients from the ANRS SEROCO cohort. Oncoimmunology (2013) 2:e23472. 10.4161/onci.23472 23802087 PMC3661172

[102] Mantovani S Mele D Oliviero B Barbarini G Varchetta S Mondelli MU. NKp30 isoforms in patients with chronic hepatitis C virus infection. Immunology (2015) 146:234–42. 10.1111/imm.12495 26094914 PMC4582964

[103] Jabrane-Ferrat N Siewiera J. The up side of decidual natural killer cells: new developments in immunology of pregnancy. Immunology (2014) 141:490–7. 10.1111/imm.12218 24256296 PMC3956423

[104] Ashkar AA Croy BA. Functions of uterine natural killer cells are mediated by interferon gamma production during murine pregnancy. Semin Immunol (2001) 13:235–41. 10.1006/smim.2000.0319 11437631

[105] Siewiera J Gouilly J Hocine HR Cartron G Levy C Al-Daccak R Natural cytotoxicity receptor splice variants orchestrate the distinct functions of human natural killer cell subtypes. Nat Commun (2015) 6:10183. 10.1038/ncomms10183 26666685 PMC4682172

[106] Shemesh A Tirosh D Sheiner E Tirosh NB Brusilovsky M Segev R First trimester pregnancy loss and the expression of alternatively spliced NKp30 isoforms in maternal blood and placental tissue. Front Immunol (2015) 6:189. 10.3389/fimmu.2015.00189 26082773 PMC4450658

[107] Shemesh A Brusilovsky M Hadad U Teltsh O Edri A Rubin E Survival in acute myeloid leukemia is associated with NKp44 splice variants. Oncotarget (2016) 7:32933–45. 10.18632/oncotarget.8782 27102296 PMC5078064

[108] Shemer-Avni Y Kundu K Shemesh A Brusilovsky M Yossef R Meshesha M Expression of NKp46 splice variants in nasal lavage following respiratory viral infection: domain 1-negative isoforms predominate and manifest higher activity. Front Immunol (2017) 8:161 10.3389/fimmu.2017.00161 28261217 PMC5309248

